# Physical function continues to improve when clinical remission is sustained in rheumatoid arthritis patients

**DOI:** 10.1186/s13075-015-0719-x

**Published:** 2015-08-11

**Authors:** Helga Radner, Farideh Alasti, Josef S. Smolen, Daniel Aletaha

**Affiliations:** Department of Internal Medicine III, Division of Rheumatology, Medical University Vienna, Waehringer Guertel 18-20, A-1090 Vienna, Austria

## Abstract

**Introduction:**

To investigate the course of functional status assessed by health assessment questionnaire (HAQ) in rheumatoid arthritis (RA) patients with sustained clinical remission (REM).

**Methods:**

In recent RA clinical trials, we identified patients with subsequent visits of ≥24 weeks in clinical REM according to the disease activity score using 28-joint counts including C-reactive protein (DAS28) (≤2.6), or simplified disease activity index (SDAI) (≤3.3). Area under the curve (AUC) and mean HAQ scores throughout the time in sustained REM were compared using *t* test, analyses of variance (ANOVA) and adjusted general linear modeling (GLM) with repeated measures. In Cox regression analyses, the time to regain full physical function was modeled. Sensitivity analyses were performed in patients of sustained SDAI low disease activity (LDA; SDAI ≤11).

**Results:**

A total of 610 out of 4364 patients achieved sustained DAS28 REM (14 %) and 252 SDAI REM (5.8 %). ANOVA testing for linear trend showed significant decrease of mean HAQ from week 0 (start of REM) to week 24, regardless of REM criteria used. AUC of HAQ throughout 24 weeks of REM was higher in DAS28 compared to SDAI REM (*p* ≤0.01). GLM adjusting for covariates showed significant decrease of monthly HAQ scores from week 0 to 24 (DAS28: 0.276, 0.243, 0.229, 0.222, 0.219, 0.209 to 0.199; *p* = 0.0001; SDAI: 0.147, 0.142, 0.149, 0.129, 0.123, 0.117 to 0.114; *p* = 0.029). Similarly, a decrease of HAQ over time was found in patients of sustained SDAI LDA. In DAS28 REM, the chance of regaining full physical function was higher for female (hazard ratio HR [95 % confidence interval]: 1.41 [1.13–1.76]) and early RA patients (disease duration ≤2 years: HR 1.29 [1.01–1.65]); in SDAI REM no significant differences were found.

**Conclusions:**

Physical function continues to improve if the target of REM or LDA is sustained. The stringency of the remission criteria determines achievement of the best possible functional improvement.

**Electronic supplementary material:**

The online version of this article (doi:10.1186/s13075-015-0719-x) contains supplementary material, which is available to authorized users.

## Introduction

The introduction of new treatment strategies including synthetic and biological agents has facilitated effective management of rheumatoid arthritis (RA), leading to better outcomes than seen in earlier years [1–5]. Clinical remission (REM) has become a widely accepted treatment goal, at least in patients with early disease, because patients in REM show higher quality of life, better physical function and work capacity, even when compared to low disease activity [6, 7]. Various definitions of REM are available exerting different levels of stringency [8, 9]. In 2011 the American College of Rheumatology (ACR) and the European League against Rheumatism (EULAR) provided two new definitions of REM, showing good predictive validity for radiographic damage and physical function [10]. Reaching remission, however, is only the first important goal in a targeted treatment approach, while the maintenance of remission is yet another important subsequent step. It is evident from previous studies that progression of joint damage decreases and ultimately halts with increasing duration of REM [11].

Even more relevant than inhibiting structural damage is the prevention of persistent functional disability in patients with RA. Disability affects patients’ overall well-being at any point in time and correlates with important long-term disease consequences, such as inability to work or mortality [12–15]. While it had been demonstrated that patients in clinical remission show better functional outcomes compared to more active disease states [7, 16], it is unknown if functional capacity further improves or is just sustained if the state of remission is maintained over time.

Therefore, the aim of this study was to investigate the course of physical function in patients with sustained clinical remission, using a large sample of patients included in several clinical trials.

## Methods

### Databases

We contacted the sponsors of pivotal clinical trials in patients with RA in which newly introduced tumor necrosis factor inhibitors (TNFi) with or without methotrexate (MTX) or synthetic disease-modifying antirheumatic drugs (DMARDs), in particular MTX, sulfasalazine or leflunomide (LEF) comprised one of the treatment arms. We were kindly provided with a random 80–90 % sample of patients studied, including all relevant data for the present investigation, from the baseline to the last visit of each study (1 or 2 years) by agreement with the sponsors including Abbvie, Amgen, Janssen, Pfizer and Sanofi and had permission to use these data for analyses and publication of the results. These trials were: the ASPIRE trial of infliximab plus MTX versus MTX alone in MTX-naïve patients with early RA of 3 years or less; [17] the ATTRACT trial of infliximab and MTX versus placebo and MTX in patients with inadequate prior response to MTX; [18] the PREMIER trial on adalimumab versus MTX versus the combination of the two in patients with early RA, who did not have previous MTX treatment; [1] the DE019 trial on adalimumab plus MTX versus placebo + MTX in patients with inadequate response to MTX; [19] the Early RA (ERA) trial of etanercept versus MTX; [20] the TEMPO trial, which compared etanercept monotherapy, etanercept plus MTX, and MTX monotherapy in patients with established RA of 6 months to 20 years duration; [5] and trials of leflunomide (‘LEF’ trials) comparing leflunomide to sulfasalazine or MTX [21–24]. There were no significant differences in demographic and clinical data between the randomly provided samples and the full trial populations.

All patients had active RA at study entry, with requirements for at least 6–10 swollen joints and at least 6–12 tender joints. With the exception of the ASPIRE trial, elevations in acute phase reactants were also required (CRP ≥1.5–2.0 mg/dL or erythrocyte sedimentation rate, ESR, ≥28 mm/h). Functional scores based on the health assessment questionnaire (HAQ) disability index were available in all trials at multiple time points. Patient demographics have been presented in the respective publications. Ethical approval was obtained for each individual study, as referenced in the respective publications, by the institutional review board at each study center and the studies were carried out in accordance with the Helsinki Declaration.

### Outcome variables

Separately for each trial dataset, we identified all study visits with available measurements of the simplified disease activity index (SDAI), the disease activity score using 28-joint counts including C-reactive protein (DAS28), as well as HAQ scores. For each patient, regardless of treatment arm, we investigated the presence of REM at all study visits by applying the respective cut-points (SDAI ≤3.3 or DAS28 ≤ 2.6). As patients can move in and out of a state of REM over time, we next identified all connected (subsequent) visits in REM to identify sustained REM periods. If a composite measure of disease activity was missing for one observation between two observed visits in REM, we assumed that the patient had also been in REM at the missing visit. If it was missing for more than one visit in the course of follow-up, then the respective remission period was not considered to be further sustained. Then, for each patient we obtained HAQ scores from the longest period in REM, which was not necessarily the first period in REM. We labeled the first visit of that period as week 0 of the respective remission segment; all subsequent visits were related to that baseline in weeks. In this way we could summarize REM periods of different patients even if they occurred at different time points during the trial(s). Only patients with at least three subsequent visits in REM over a period of at least 24 weeks throughout the course of the trials were used for further analyses of their HAQ score changes. Since the underlying trials had different visit intervals, we interpolated, if needed, HAQ scores of each patient for weeks 0, 4, 8, 12, 16, 20, and 24. This was done to allow us to depict the courses of HAQ in sustained remission across a large group of patients.

### Statistical analyses

To assess the course of physical function over time in sustained REM, we calculated mean monthly HAQ scores and compared them across time, using analyses of variance (ANOVA) testing for linear trend. Furthermore, the area under the curve (AUC) of HAQ scores over time in sustained remission was calculated. All analyses were performed separately for patients in early (disease duration ≤2 years) and established (disease duration >2 years) RA. Individuals regaining full physical function (defined as HAQ = 0) were identified and their proportion was plotted over time in sustained REM.

To adjust for factors associated with physical function, we extended ANOVA to general linear models (GLM) with repeated measures, using HAQ as dependent variable. Covariates included in the model were: age, disease duration, gender, serological status (rheumatoid factor [RF]), radiographic damage (modified total Sharp score [mTSS]), time from randomization to first visit in sustained remission, HAQ, SDAI or DAS28 at the onset of sustained REM, and change of disease activity (SDAI or DAS28) from week 0 to week 24 in sustained REM. To account for treatment regimes, patients were grouped according to the drug regimen (synthetic DMARD monotherapy: MTX, LEF or sulfasalazine; TNFi monotherapy: adalimumab or etanercept; TNFi combination therapy with a synthetic DMARD: adalimumab, etanercept, or infliximab plus MTX), and the treatment grouping variable was included in the model. Estimated marginal means (EMM) were calculated, by setting the continuous covariates to their cohort means.

Hypothesizing that physical function would also continue to improve if patients were in sustained low disease activity (LDA), we performed a sensitivity analysis in a subgroup of DAS28 REM patients who were in sustained SDAI LDA (SDAI ≤11) throughout the observation period.

In Cox regression analyses the time to regain full physical function, defined by achievement and sustainment of HAQ = 0, was modeled. Covariates as described above were entered stepwise in the model; variables with a significance level higher than 0.1 were removed, as they did not contribute to the model. In supplementary analyses we also looked at the achievement of good physical function (HAQ ≤0.5) [25] and repeated Cox regression analyses.

## Results

### Patient characteristics

The cohort studied comprised 4364 patients with RA; throughout the follow-up of these patients, 610 of them (14.0 %) had a period of sustained remission of at least 24 weeks defined by DAS28, and 252 patients (5.8 %) had sustained REM as defined by SDAI. Baseline characteristics of all patients at randomization of the total cohort as well as of patients identified as in sustained REM by the DAS28 or by the SDAI are depicted in Table 1. Patients achieving sustained REM showed lower levels of disease activity, higher frequencies of RF, and shorter duration of RA (*p* <0.05 for all) compared to the total study population. No significant differences of baseline characteristics were found between patients achieving sustained DAS28 and those in SDAI REM, except that patients achieving SDAI REM had significantly shorter duration of RA (*p* = 0.001; Table 1). Disease activity variables at the start of sustained DAS28 and SDAI REM are shown in Table 2. Mean HAQ scores as well as other measures of disease activity at the start of sustained SDAI REM were significantly lower than those at the start of sustained DAS28 REM (*p* <0.01 for all variables except CRP). On average, the first period of sustained REM defined by DAS28 started at 26.2 ± 20.2 weeks after randomization, while sustained REM according to SDAI started at 31.2 ± 22.1 weeks (*p* = 0.004). Over the time in sustained REM defined by DAS28, an increasing proportion of these patients also achieved SDAI REM (41.8 % at the start of sustained DAS28 REM and 59.2 % at week 24 of sustained DAS28 REM); independent of the definition of REM, all measures of disease activity except CRP decreased significantly from week 0 to week 24 in sustained remission, (*p* <0.01 for all variables; Table 2).

**Table 1:** Patient characteristics at study entry: overall patient cohort (first column), patients in sustained clinical remission of 24 weeks or longer according to the DAS28 (second column) or SDAI definition (last column) of remission

	Total cohort	DAS28 REM	SDAI REM	*p* value total cohort vs. DAS28 REM	*p* value total cohort vs. SDAI REM	*p* value DAS28 REM vs. DAS28 REM
Total number of patients (%)	4364	610 (14.0 %)	252 (5.8 %)			
Female (%)	73.90 %	64.5 %	62.3 %	<0.01	<0.01	0.56
Ethnicity (white %)	76.5 %	75.5 %	85.3 %	0.37	0.20	0.52
Age (years)	52.9 ± 12.8	49.8 ± 13.5	49.5 ± 13.2	<0.01	<0.01	0.81
Disease duration (years)	4.1 ± 6.0	2.5 ± 4.1	1.6 ± 2.8	<0.01	<0.01	0.01
Rheumatoid factor pos (%)	74.5 %	74.9 %	78.2 %	0.88	0.20	0.3
Disease activity score using 28-joint counts including CRP (DAS28)	6.5 ± 1.1	5.53 ± 1.1	5.6 ± 1.0	<0.01	<0.01	0.73
Simplified disease activity index (SDAI)	44.1 ± 14.9	37.6 ± 14.1	37.7 ± 13.8	<0.01	<0.01	0.97
Health assessment questionnaire (HAQ)	1.5 ± 0.65	1.24 ± 0.64	1.26 ± 0.63	<0.01	<0.01	0.72
Swollen joint count 28 joints (SJC28)	13.2 ± 6.3	11.2 ± 5.7	10.8 ± 5.2	<0.01	<0.01	0.39
Tender joint count 28 joints (TJC28)	15.4 ± 6.9	12.8 ± 6.5	12.8 ± 5.9	<0.01	<0.01	0.88
Visual analogue scale for pain (VAS pain in mm)	48.2 ± 29.9	51.1 ± 24.5	50.8 ± 24.0	0.02	0.18	0.87
Patient global assessment of disease activity (PGA in mm)	61.1 ± 22.0	54.3 ± 23.8	52.8 ± 25.2	<0.01	<0.01	0.41
Evaluator global assessment of disease activity (EGA in mm)	62.3 ± 18.0	57.5 ± 20.0	58.1 ± 20.0	<0.01	<0.01	0.76
C-reactive protein (CRP)	3.2 ± 3.8	2.6 ± 3.1	2.8 ± 3.3	<0.01	0.14	0.29
Radiographic damage (modified Sharp score)	28.8 ± 42.1	18.3 ± 26.3	15.3 ± 20.7	<0.01	<0.01	0.11
Treatment regime (%)						
Synthetic DMARD monotherapy	40.4 %	25.4 %	20.2 %	<0.01	<0.01	0.11
TNFi monotherapy	37.5 %	43.0 %	46.8 %	0.01	<0.01	0.33
TNFi combination therapy	20.6 %	31.6 %	32.9 %	<0.01	<0.01	0.75

Values are given for means ± standard deviation, unless indicated otherwise; *p* values were obtained by comparing characteristics of the total cohort with patients in DAS28 REM or SDAI REM and comparing patients in DAS28 REM and SDAI REM. *REM* remission, *DMARD* disease-modifying antirheumatic drugs, *TNFi* tumor necrosis factor inhibitor

**Table 2:** Patients characteristics at first visit and week 24 in sustained remission for patients in sustained clinical remission of 24 weeks or longer according to the DAS28 and SDAI definition of remission

	DAS28 REM			SDAI REM		
	Week 0	Week 24	*p*	Week 0	Week 24	*p*
Disease activity score using 28-joint counts including CRP (DAS28)	2.1 ± 0.3 (1.21–2.6)	1.9 ± 0.3 (1.01–2.59)	*P* <0.01	1.8 ± 0.28 (1.21–2.72)	1.7 ± 0.2 (1.21–2.72)	*P* <0.01
Simplified disease activity index (SDAI)	4.3 ± 2.8 (0.3–16.1)	3.2 ± 2.5 (0.1–18.2)	*P* <0.01	1.9 ± 0.9 (0.1–3.3)	1.3 ± 0.8 (0.1–3.24)	*P* <0.01
Health assessment questionnaire (HAQ)	0.28 ± 0.4 (0–2.25)	0.20 ± 0.4 (0–1.92)	*P* <0.01	0.15 ± 0.3 (0–2.25)	0.11 ± 0.3 (0–1.75)	*P* <0.01
Swollen joint count 28 joints (SJC28)	1.3 ± 2.2 (0–15)	0.8 ± 2.0 (0–17)	*P* <0.01	0.3 ± 0.5 (0–2)	0.2 ± 0.4 (0–2)	*P* <0.01
Tender joint count 28 joints (TJC28)	0.4 ± 0.7 (0–3)	0.2 ± 0.5 (0–3)	*P* <0.01	0.2 ± 0.5 (0–2)	0.1 ± 0.4 (0–2)	*P* <0.01
Visual analogue scale for pain (VAS pain in mm)	8.8 ± 10.9 (0–80)	6.9 ± 8.5 (0–64)	*P* <0.01	4.4 ± 6.1 (0–53)	3.0 ± 4.0 (0–24)	*P* <0.01
Patient global assessment of disease activity (PGA in mm)	11 ± 11.8 (0–70)	8.8 ± 9.6 (0–57)	*P* <0.01	4.5 ± 5.4 (0–27)	3.5 ± 4.9 (0–30)	*P* <0.01
Evaluator global assessment of disease activity (EGA in mm)	10.2 ± 10.3 (0–92)	7.7 ± 8.9 (0–93)	*P* <0.01	4.4 ± 4.7 (0–24)	3.1 ± 3.7 (0–17)	*P* <0.01
C-reactive protein (CRP, mg/dL)	0.50 ± 0.6 (0.1–9.4)	0.52 ± 0.5 (0.1–4.3)	*P* = 0.46	0.49 ± 0.4 (0.1–2.92)	0.50 ± 0.4 (0.1–3.8)	*P* = 0.69

Values are given for means ± standard deviation, significant differences were detected, using Student’s *t* test. Values in parentheses depict ranges. Differences between DAS28 REM and SDAI REM were significant at *p* <0.01 for all variables and time points except for CRP. *REM* remission

### Unadjusted analyses of functional course in sustained remission

**Fig. 1:**
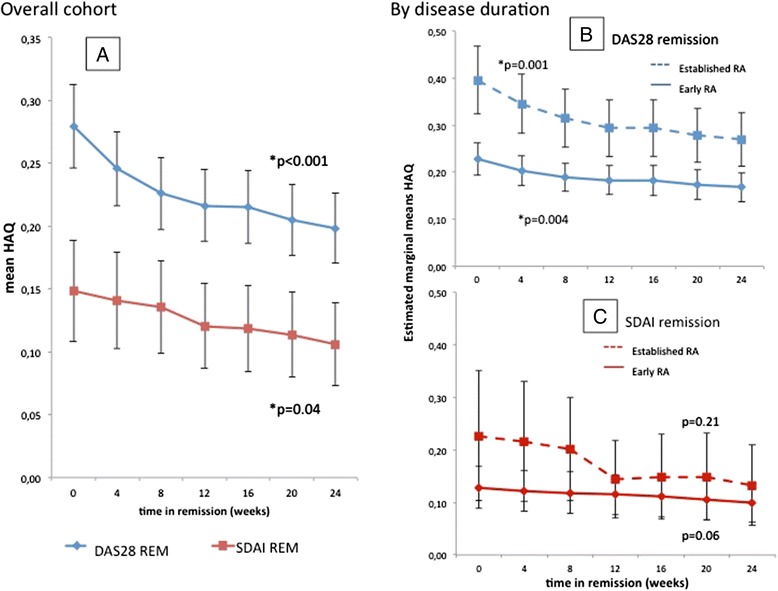
Unadjusted analyses of variance (ANOVA): **a** mean values of health assessment questionnaire (HAQ) plotted over time in sustained clinical remission defined by DAS28 (*blue line*) or SDAI (*red line*) showing a decrease over time. Stratification of the cohort in early (disease duration ≤2 years, *full line*) and established RA patients (*dotted line*) in sustained remission defined by (**b**) DAS28 or (**c**) SDAI (*p* values testing for linear trend; asterisk indicates significance). *DAS28* disease activity score using 28-joint counts including CRP, *RA* rheumatoid arthritis, *SDAI* simplified disease activity index

Using ANOVA we found a significant improvement of HAQ scores over time in sustained DAS28 REM (*p* <0.001; testing for linear trend) of 28.6 % from a mean of 0.28 ± 0.4 at onset of the remission period (week 0) to 0.20 ± 0.3 after 24 weeks (Fig. 1a; *blue line*). In SDAI REM we also found a significant linear trend (*p* = 0.04). Mean HAQ scores were significantly lower in SDAI REM compared to DAS28 REM with significantly lower AUC of HAQ in SDAI REM compared to DAS28 REM (*p* <0.001; Fig. 1a). In fact, after 24 weeks of sustained DAS28 REM, HAQ scores were on average still higher (0.20 ± 0.4) than they had already been at the onset of SDAI-based REM (week 0: 0.15 ± 0.3). When we split patients according to their disease duration, those with early RA in DAS28 REM had significantly lower AUC of HAQ compared to those with established RA (*p* <0.001; Fig. 1b). There was a significant decrease of HAQ over time for both groups (Fig. 1b; ANOVA testing for linear trend: *p* = 0.004 for early RA; *p* = 0.001 for established RA). For SDAI REM, no significant differences of AUC of HAQ between early and established RA were found (*p* = 0.22). In addition, no significant change of mean HAQ scores over time in SDAI REM were seen within the early and established RA groups (Fig. 1c; ANOVA testing for linear trend: *p* = 0.21 and *p* = 0.06 respectively). However, the mean HAQ scores at the start of sustained SDAI REM were already quite low irrespective of disease duration, leaving little room for further statistically significant improvement until week 24.

In sensitivity analyses we looked at the course of function in patients with complete data without interpolation of HAQ showing similar results and a decrease of HAQ over time in remission (Figure S1 in Additional file 1).

### Adjusted analyses of the course of physical function in sustained remission

**Fig. 2:**
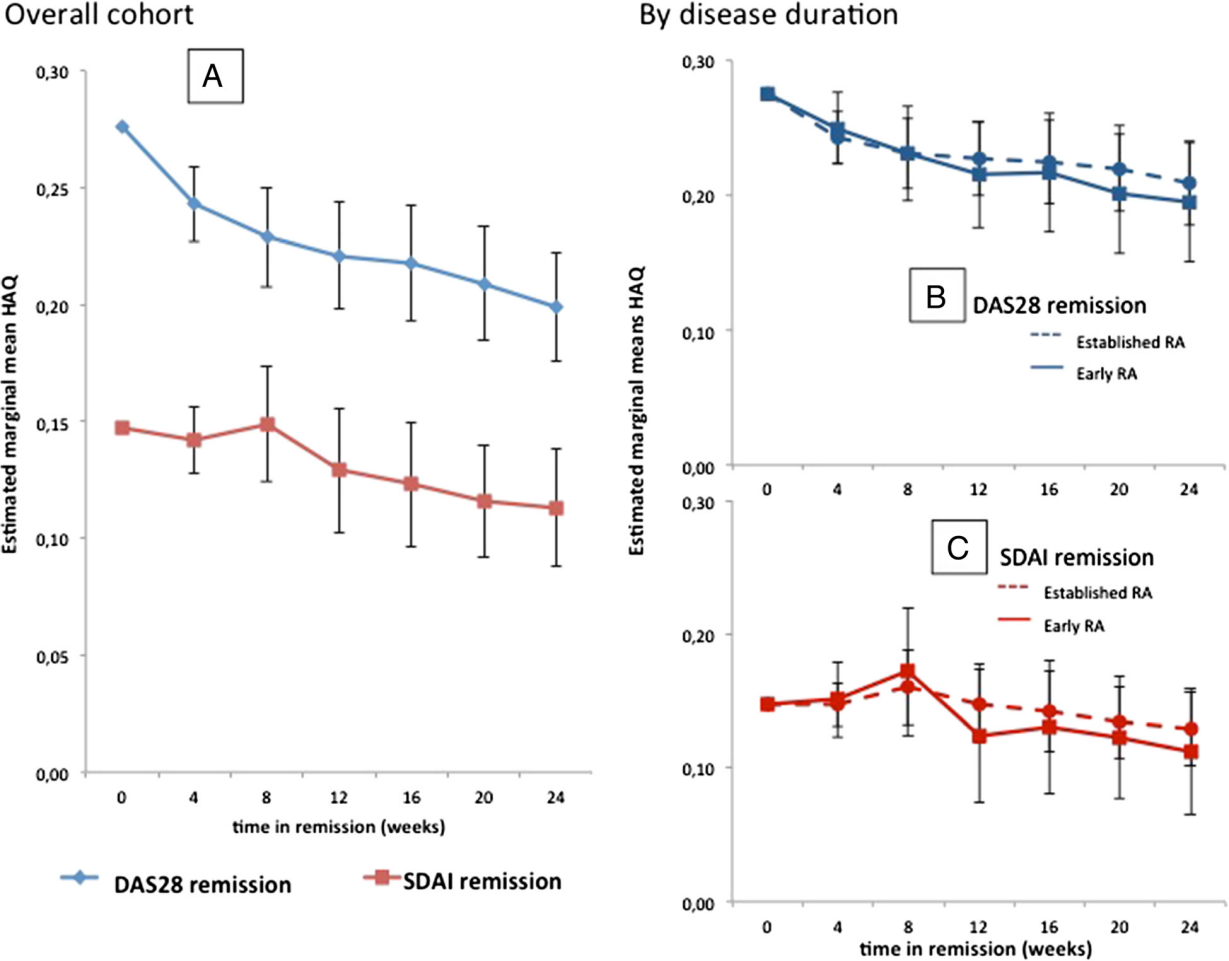
Physical disability decreases over time in sustained remission after adjusting for covariates. **a** For the DAS28 (*blue line*), the graphs depict the means of the health assessment questionnaire (HAQ) considering all covariates, i.e., estimating for a female, seropositive patient at age 49.6 years, disease duration of 2.4 years, time to sustained REM of 26.8 weeks, a change of DAS28 from week 0 to week 24 in REM of 0.17, and a DAS28 of 2.07 and a HAQ of 0.28 at first REM visit, as well as a modified total Sharp score (mTSS) of 18.35; for the SDAI (*red line*) the means were estimated for a female, seropositive patient at age 49.6, with a disease duration of 1.5 years, a time to sustained REM of 31.7 weeks, a change of SDAI from week 0 to week 24 in REM of 0.54, and an SDAI of 1.87 and HAQ of 0.15 at the first REM visit, as well as a mTSS Sharp score of 15.55. Mean as estimated separately for early RA (disease duration ≤2 years; *dotted line*) and established RA (disease duration >2 years; *full line*) patients in (**b**) DAS28 REM and (**c**) SDAI REM showed a significant decrease of HAQ scores over time in sustained REM, but no differences between early and established RA patients. *DAS28* disease activity score using 28-joint counts including CRP, *RA* rheumatoid arthritis, *REM* remission, *SDAI* simplified disease activity index

After adjusting for covariates in GLM repeated measures, we found a significant change of HAQ scores over time in patients with sustained DAS28 REM (*p* = 0.02). Mean HAQ scores estimated for each time point during sustained REM showed a significant decrease from week 0 to week 24 (0.276, 0.243, 0.229, 0.222, 0.219, 0.209 to 0.199 at 4-weekly assessments; *p* <0.01; i.e., a further improvement of 28.7 %; Fig. 2a, *black line*). Similar results were obtained when defining REM using SDAI-based REM: mean HAQ scores were 0.147, 0.142, 0.149, 0.129, 0.123, 0.117, and 0.114, respectively, for the different time points; i.e., a further improvement of 23.1 %; *p* = 0.029; Fig. 2a*red line*). Using either REM criteria, no significant effect of disease duration (early RA ≤2 years versus established RA >2 years) was found (Fig. 2, panel b DAS28 REM *p* = 0.57; panel c SDAI REM *p* = 0.70).

In a sensitivity analysis we looked at a subgroup of patients in DAS28 REM who were in sustained SDAI LDA (n = 159) over 24 weeks. We found a similar significant decrease of mean HAQ over time in sustained SDAI LDA in unadjusted ANOVA (testing for linear trend *p* <0.001) and GLM adjusting for covariates (Figure S2 in Additional file 2).

### Achievement of full physical function during sustained remission

**Fig. 3:**
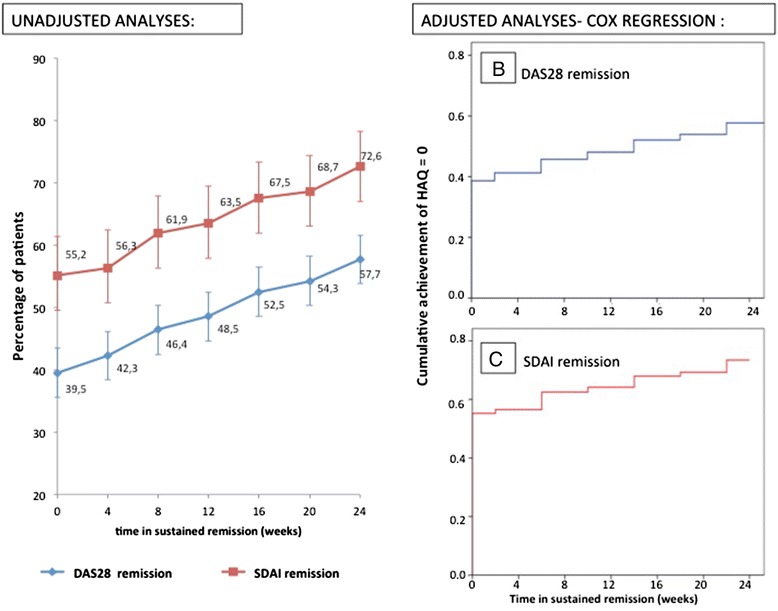
Recovery of full physical function (HAQ = 0) during the time in sustained remission. **a** Unadjusted analyses: percentage (95 % confidence interval) of patients regaining full physical function in REM defined by DAS28 (*blue line*) and SDAI (*red line*). Adjusted Cox regression analyses adjusted for age, gender, radiographic damage, disease duration, disease activity at week 0 in REM, change of disease activity from week 0 to week 24 in REM, and time from randomization until sustained REM. Survival curves were plotted separately for (**b**) DAS28 and (**c**) SDAI REM. *DAS28* disease activity score using 28-joint counts including CRP, *HAQ* health assessment questionnaire, *REM* remission, *SDAI* simplified disease activity index

Over time in sustained REM, 57.7 % of the patients in DAS28 REM regained full physical function (HAQ = 0). This proportion was significantly higher in patients with sustained SDAI REM (72.6 %, *p* <0.001). The percentage of patients regaining full physical function is depicted in Fig. 3a, separately for patients in DAS28 and SDAI REM.

In a Cox regression model predicting recovery of full physical function over time in sustained REM, we included additional explanatory variables. In DAS28 REM the hazard ratio (HR; 95 % confidence intervals [CIs]) for regaining full physical function was 1.41 (1.13–1.76) times higher for female compared to male patients, and 1.29 (1.01–1.65) times higher for early RA patients compared to those with established RA (Table 3, left column; Fig. 3, panel b). For patients in sustained SDAI REM, only age significantly contributed to the model, with decreasing HR of regaining full physical function with increasing age (HR 0.98; 95 % CI 0.97–0.99; (Table 3, right column; Fig. 2, panel c). In DAS28 as well as SDAI REM treatment regimen and seropositivity were found to not significantly contribute to the achievement of full physical function and therefore were excluded from the final models.

**Table 3:** Cox regression to model the time to regain full physical function, defined by a sustained health assessment questionnaire (HAQ) score of zero within time in sustained remission

Variables included in the model	DAS28 remission			SDAI remission		
	HR	95 % CI	Sig	HR	95 % CI	Sig
Age (years)	0.98	0.97–0.99	<0.01	0.98	0.97–0.99	0.01
Early RA (≤2 years disease duration)	1.29	1.01–1.65	0.049	Removed from final model		
Gender (female)	1.41	1.13–1.76	0.002	Removed from final model		
Time from randomization until sustained remission (weeks)	0.99	0.98–0.99	0.014	Removed from final model		
DAS28/SDAI at week 0 in sustained remission	0.36	0.23–0.57	<0.01	Removed from final model		
Change DAS28/SDAI from week 0 to week 24 in sustained remission	1.67	1.16–2.40	0.006	Removed from final model		

Table depicts hazard ratios (HR) and 95 % confidence interval (95 % CI) of all covariates included in the model, separately for patients in sustained DAS28 and SDAI defined remission. *DAS28* disease activity score using 28-joint counts including CRP, *SDAI* simplified disease activity index, *HR* hazard ratio, *CI* confidence interval, *RA* rheumatoid arthritis

In sensitivity analyses we also looked at the recuperation of good physical function (HAQ ≤0.5), showing again statistically higher percentages in patients with SDAI REM compared to DAS28 REM (93.3 % versus 85.6 %, *p* = 0.001; Figure S3 panel A in Additional file 3). When dividing patients into subgroups by disease duration, 90.4 % of early RA patients achieving SDAI REM regained good physical function already at week 0 of REM compared to 80.8 % with established disease (Figure S3 panel B in Additional file 3). After adjusting for covariates in Cox regression analyses predicting good function, no differences in HR between early and late RA patients could be observed in DAS28 REM or SDAI REM (Figure S3 panel C and D in Additional file 3).

## Discussion

In our study we were able to show that physical function continues to improve over time when remission is maintained. Thus, improvement of physical function does not reach a maximum extent with the first achievement of a good clinical state, but only with maintenance of this condition. This is reminiscent of data showing that halt of joint damage depends on maintenance of a good clinical outcome such as REM [26]. For joint damage it was implied that, despite overall reduction in progression rates already at the time when REM was first achieved, there was still a carry-over effect of destructive mechanisms beyond this time point of an initial REM state [11]. Along similar lines, one may conclude that functional limitations that were a consequence of the disease process also need time to recover after the disease process has subsided on therapy.

The other finding is the fact that the stringency of the REM definition itself not only determines the functional state of those reaching REM, but also – as a consequence – the degree of further functional improvement if REM is sustained. This is apparent when comparing the functional courses of patients in DAS28 REM and SDAI REM: it is well known that most core set variables are significantly higher at the onset of DAS28 REM, than as the stringent SDAI REM; this apparently has led to the fact that HAQ scores in REM were not only higher in DAS28 REM, but also HAQ improvement in sustained REM was more clearly seen when using the DAS28 < 2.6 definition than the SDAI definition. HAQ scores at the time of the initial visit in SDAI REM had already reached a mean value of 0.15. Thus, the stringency of REM criteria determines the extent of recovery of physical ability, and the more stringent the criteria, the larger the extent of functional improvement which had already taken place during the time before reaching that state. Indeed, with maintenance of REM, whichever way defined, there is also continuing improvement of the respective disease activity scores so that after 6 months of sustained REM the mean values of most individual disease activity measures are significantly lower than at the start of the sustained REM period. A sensitivity analysis performed in patients with sustained SDAI LDA underlines the association of simply maintaining a low disease activity state as an alternative good treatment outcome with improvement of physical function over time.

Previous studies have suggested an association between time in REM and functional or radiographic outcomes; [11, 16] the shorter the period of REM, the more likely some radiographic progression was present. Joint damage can serve as a surrogate marker for irreversible impairment of physical function [27, 28]. Furthermore, also the level of disease activity in REM is important. Even when already reaching REM, depending on the definition of REM, residual joint inflammation might be observed, which could be an explanation for structural deterioration in RA patients [29, 30]. Indeed, in our cohort, patients in REM defined by DAS28 showed up to 17 swollen joints, compared to patients in SDAI REM, where a maximum of two swollen joints was present. Thus, the more stringent the REM criteria are, the higher the chance for good functional and structural outcomes, as less clinical abnormalities are present and fewer subclinical pathologies are detectable [31]. However, it must be borne in mind that structural progression in REM by any means is relatively small, and that other factors contribute to the observable improvement in function. Even after adjusting for covariates such as age, disease duration or radiographic damage, and finally also disease activity itself (which also decreased in sustained remission) such improvement in physical function was observed. Several explanations for this phenomenon come to mind. First, it is likely that mobility of previously inflamed joints, muscle wasting related to disease activity, and systemic features, such as fatigue and malaise need time to fully restore after disease activity has stopped. Second, however, it is also possible that even the small residual disease activity in remission contributes to some functional impairment and that the observed improvement of this residual disease activity over time in sustained REM has an impact on physical function due to the continuing decrease of the residual low degrees of pain and tenderness. The significantly better physical function in sustained SDAI-defined REM compared to DAS28 is well in line with this notion.

In our study we found an average improvement of HAQ of 0.08 within 24 weeks of sustained DAS28 REM or respective 0.04 in SDAI REM. Even though these changes may seem small in their extent, it has to be emphasized that patients in REM are already in very good functional status to start with. On the group level in this population, the relative further improvement was between a quarter and a third of the initial HAQ assessment. Almost 20 % of the patients regained full physical function within sustained REM. Also, on an individual patient level, improvement of HAQ showed wide variability with change of HAQ of up to 2.1 in DAS REM and 0.8 in SDAI REM, with a large number of patients experiencing further improvement in sustained remission beyond the minimal clinically important difference of 0.2 [32, 33]. Furthermore, as the HAQ scale shows nonlinearity, a change from 1.6 to 1.4 may not be the same as changes from 0.4 to 0.2. Indeed, it was shown previously that the threshold of symptomatic difference is smaller if patients are less disabled [34].

Several limitations of our study need to be addressed: first, the evaluated trials had partly different visit intervals. Since we interpolated to monthly assessments, fluctuation of disease activity might have been missed and some patients assumed to be in sustained remission while in fact they were not. By requiring at least three subsequent visits in REM, and 24 weeks of observation time, it is unlikely that this limitation applied to a significant number of patients. Second, the development of physical disability is a multifactorial process with different aspects and causes, some of which are RA related, while others are related to other factors in the patient’s life such as comorbidities or psychological status [35, 36]. Some of these factors were not measured in our study; therefore, our analyses did not account for them. Third, we employed DAS28, since we had CRP available in all trials, but ESR was missing in some of them; however, in a sensitivity analysis using DAS28-ESR we obtained very similar results.

A strength of our study is the use of a large sample of patients included in recent large-scale randomized controlled RA clinical trials. Due to the respective inclusion and exclusion criteria, patients were homogeneous, which allows the best possible assessment of different associations. Nevertheless, due to this preselected conditions, generalization of findings might be limited. In addition, the included trials were conducted before the introduction of the new 2010 ACR/EULAR classification criteria of RA and, therefore, our conclusions may not be assignable to all RA populations.

## Conclusions

In summary, we were able to show that physical function continues to improve over time if a clinical target is reached and sustained, as shown here for a state of low disease activity or remission. The functional level at the onset of remission, as well as the ability to improve depends on the stringency of the remission criteria used. These data call for and support the continuation of following a treatment goal of sustained clinical remission. This will allow for all patients to regain their best physical function possible.

## Additional files

Additional file 1: Figure S1. Unadjusted analyses of variance (ANOVA) in patients with complete data on HAQ: mean values of the health assessment questionnaire (HAQ) plotted over time in sustained clinical remission (REM) defined by DAS28 (n = 132; *blue line*) or SDAI (n = 26; *red line*) showing a decrease over time. ANOVA testing for linear trend was significant in patients achieving DAS28 REM (*p* = 0.02), but not in SDAI REM (*p* = 0.41). (JPEG 32 kb) Additional file 2: Figure S2. Physical disability decreases over time in sustained low disease activity (LDA). (A) Unadjusted analyses of variance (ANOVA): mean values of the health assessment questionnaire (HAQ) plotted over time in sustained LDA defined by the simplified disease activity index (SDAI ≤11) showing a decrease over time (*p* <0.001 ANOVA testing for linear trend). (B) Adjusted analyses: estimated marginal means of HAQ considering all covariates, i.e., estimating for a female, seropositive patient at age 50.2 years, disease duration of 2.1 years, time to sustained LDA of 21.6 weeks, a change of SDAI from week 0 to week 24 of 3.2, a SDAI of 5.0 and a HAQ of 0.28 at first LDA visit, as well as a modified total Sharp score (mTSS) of 19.6. (JPEG 46 kb) Additional file 3: Figure S3. Regain of good physical function (HAQ ≤0.5) during the time in sustained remission. Unadjusted analyses: percentage (95 % confidence interval) of patients regaining good physical function in remission defined by DAS28 (*blue line*) and SDAI (*red line*) for (A) the total cohort and (B) separately for early RA (*full line*) and established RA (*dotted line*). Adjusted Cox regression analyses adjusted for age, gender, radiographic damage, disease duration, disease activity at week 0 in REM, change of disease activity from week 0 to week 24 in REM, and time from randomization until sustained remission plotted separately for patients in (C) DAS28 and (D) SDAI remission. (JPEG 93 kb)

## Abbreviations

ACR: American College of Rheumatology
ANOVA: analyses of variance
AUC: area under the curve
CI: confidence interval
CRP: C-reactive protein
DAS28: disease activity score using 28-joint counts including CRP
DMARDs: disease-modifying antirheumatic drugs
EGA: evaluator global assessment of disease activity
EMM: estimated marginal means
ESR: erythrocyte sedimentation rate
EULAR: European League against Rheumatism
GLM: general linear model
HAQ: health assessment questionnaire
HR: hazard ratio
LDA: low disease activity
LEF: leflunomide
mTSS: modified total Sharp score
MTX: methotrexate
PGA: patient global assessment of disease activity
RA: rheumatoid arthritis
REM: remission
RF: rheumatoid factor
SDAI: Simplified Disease Activity Index
SJC28: swollen joint count including 28 joints
TJC28: tender joint count including 28 joints
TNFi: tumor necrosis factor inhibitors
VAS: visual analogue scale
