# Supplementary figures and images for: Physical function continues to improve when clinical remission is sustained in rheumatoid arthritis patients
Source: Arthritis Res Ther. 2015 Aug 11;17(1):203. doi: 10.1186/s13075-015-0719-x (PMC4530479; doi:10.1186/s13075-015-0719-x)

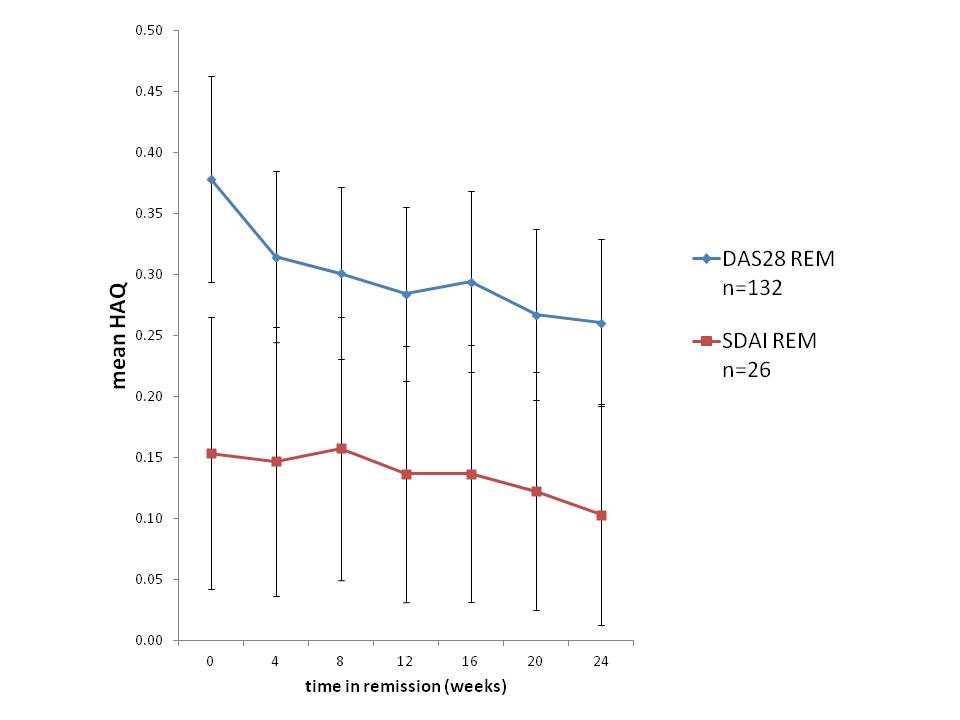

Supplement: Additional file 1: Figure S1. — Unadjusted analyses of variance (ANOVA) in patients with complete data on HAQ: mean values of the health assessment questionnaire (HAQ) plotted over time in sustained clinical remission (REM) defined by DAS28 (n = 132; blue line) or SDAI (n = 26; red line) showing a decrease over time. ANOVA testing for linear trend was significant in patients achieving DAS28 REM (p = 0.02), but not in SDAI REM (p = 0.41). (JPEG 32 kb) [file 13075_2015_719_MOESM1_ESM.jpeg]

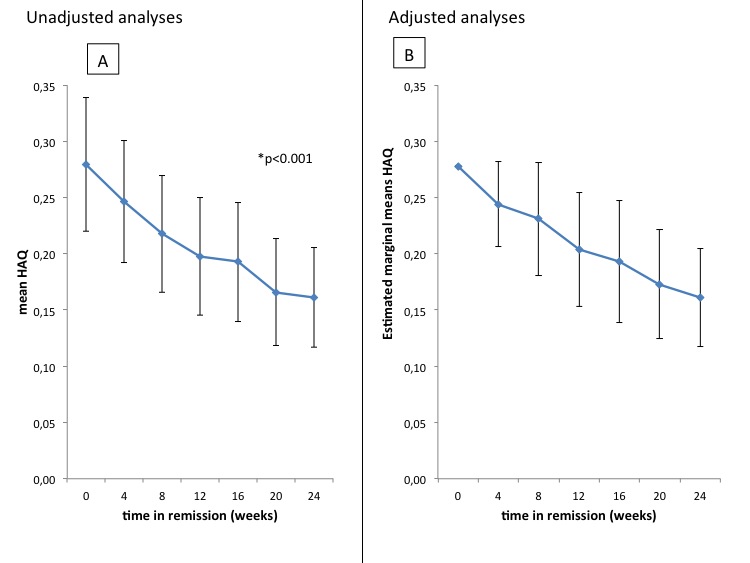

Supplement: Additional file 2: Figure S2. — Physical disability decreases over time in sustained low disease activity (LDA). (A) Unadjusted analyses of variance (ANOVA): mean values of the health assessment questionnaire (HAQ) plotted over time in sustained LDA defined by the simplified disease activity index (SDAI ≤11) showing a decrease over time (p <0.001 ANOVA testing for linear trend). (B) Adjusted analyses: estimated marginal means of HAQ considering all covariates, i.e., estimating for a female, seropositive patient at age 50.2 years, disease duration of 2.1 years, time to sustained LDA of 21.6 weeks, a change of SDAI from week 0 to week 24 of 3.2, a SDAI of 5.0 and a HAQ of 0.28 at first LDA visit, as well as a modified total Sharp score (mTSS) of 19.6. (JPEG 46 kb) [file 13075_2015_719_MOESM2_ESM.jpeg]

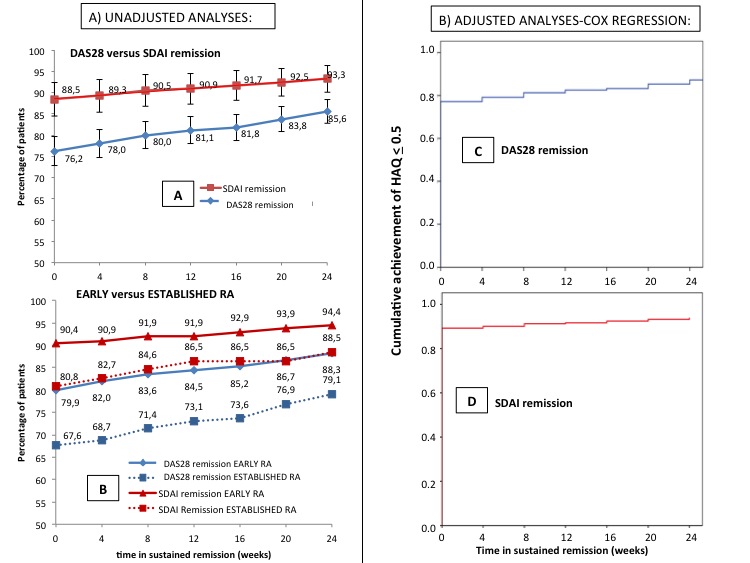

Supplement: Additional file 3: Figure S3. — Regain of good physical function (HAQ ≤0.5) during the time in sustained remission. Unadjusted analyses: percentage (95 % confidence interval) of patients regaining good physical function in remission defined by DAS28 (blue line) and SDAI (red line) for (A) the total cohort and (B) separately for early RA (full line) and established RA (dotted line). Adjusted Cox regression analyses adjusted for age, gender, radiographic damage, disease duration, disease activity at week 0 in REM, change of disease activity from week 0 to week 24 in REM, and time from randomization until sustained remission plotted separately for patients in (C) DAS28 and (D) SDAI remission. (JPEG 93 kb) [file 13075_2015_719_MOESM3_ESM.jpeg]
